# Protocol for recombinant rabies virus titration by quantitative PCR

**DOI:** 10.1016/j.xpro.2024.103567

**Published:** 2025-01-22

**Authors:** He Zhang, Xueping Gao, Xiangyu Ge, Xiao Wang, Minghui Song, Xia Zhang, Lei Jin

**Affiliations:** 1 Lingang Laboratory, Shanghai 200031, China; 2 School of Life Science and Technology, ShanghaiTech University, Shanghai 201210, China

**Keywords:** Molecular Biology, Neuroscience, Biotechnology and bioengineering

## Abstract

Preparing high-titer virus and performing accurate titer determination are critical to subsequent experiments. However, not all applied recombinant rabies viruses, such as the L-deleted virus,^1^ are equipped with fluorescent proteins for titration by fluorescence-activated cell sorting (FACS). Here, we present a quantitative reverse-transcription PCR (RT-qPCR) approach for titrating recombinant rabies virus. We describe steps for preparing standards for RT-qPCR, rabies virus genome RNA extraction, and reverse transcription of virus RNA. We then detail procedures for RT-qPCR for titration and stereotaxic rabies virus injection for titer verification.

## Highlights

• Guidance on recombinant rabies virus titering by RT-qPCR
• Instructions for recombinant rabies virus rescue, passaging, purification, and pseudotyping
• Steps for recombinant rabies virus genome RNA extraction, RT-qPCR, and calculation of titer

## Abstract (editor highlights)

Publisher’s note: Undertaking any experimental protocol requires adherence to local institutional guidelines for laboratory safety and ethics.

## Graphical abstract

**Figure:**
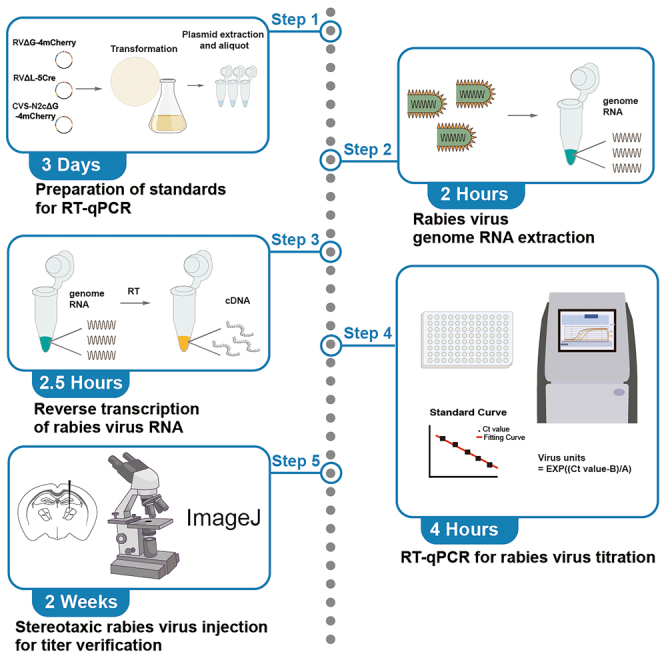

## Before you begin

The protocol below describes a technique for titrating rabies virus by RT-qPCR.

### Institutional permissions

Any experiments involving rabies virus (a Biosafety Level 2 (BSL-2) pathogen) must be performed in accordance with BSL-2 requirements and regulations. Appropriate approval from the institution performing the research and the community in which the samples were obtained is necessary prior to using this protocol.

All animal procedures were approved by the Institutional Animal Care and Use Committee (IACUC) of Lingang Laboratory in accordance with national guidelines to ensure the humane treatment of experimental animals. All mice were housed at the animal facility of the Lingang Laboratory under specific pathogen-free conditions, standard chow, water, and light/dark cycles. For animal-related procedures, it is essential that the user acquires ethical permissions from the relevant committee.

### Preparation one: Rabies virus packaging

**Timing: 2 weeks**

This section provides detailed instructions on high-titer rabies virus packaging. The cell lines used in this protocol are listed in Table 1. Specific protocols for constructing cell lines used are not described here. The condition of the incubator in this protocol is 37°C with 5% CO_2_.

1. Poly-L-lysine coating of cell culture plates.
  a. Apply 3 mL poly-L-lysine solution (0.01% poly-L-lysine 1:6 diluted with 1x DPBS) to 10 cm plate.
  b. Allow to stand for 1 h in the incubator.

***Note:*** Gently agitate to evenly distribute across the surface of the plate.

2. Cell seeding.
  a. Aspirate poly-L-lysine solution.
  b. Suspend 6 x 10^6^ cells in 8 mL culture medium (DMEM with 10% FBS and 1% antibiotic-antimycotic).
  c. Seed the cells in a poly-L-lysine-coated 10 cm cell culture plate.
  d. Incubate 18–24 h.

***Note:*** Cell number seeded at this day is very important. It is essential to have 80% cell density before subsequent plasmids transfection.

3. Rescue of rabies virus from cDNA plasmids.
  ***Note:*** The cell lines used for different recombinant rabies virus rescue, passage, and FACS titration are listed in Table 1. Please contact Lei Jin (jinlei@lglab.ac.cn) if need the cell lines or a protocol for making any cell lines in the manuscript.
  a. Prepare the transfection recipe.
    i. Dilute the plasmid DNA into 1.5 mL Opti-MEM Medium.
      ***Note:*** The amounts of plasmids (the concentration around 0.5-2 μg/μL each) used for a single 10 cm plate are listed in Tables 2 and 3 (see materials and equipment setup part).
    ii. Mix them thoroughly.
    iii. Dilute 72 μL of Lipofectamine 2000 into 1.5 mL Opti-MEM Medium in a separate tube.
      ***Note:*** For rescue the CVS-N2c strain,^2^ we found using B19G is better than using N2cG.
  b. Incubate at ∼25°C for 5 min.
  c. Add diluted DNA to diluted Lipofectamine 2000 (1:1 ratio). Incubate at ∼25°C for 20 min.
  d. During 20 min incubation, wash cells with 3 mL DPBS(1X), then replace DPBS(1X) with 5 mL Opti-MEM Medium for 10 cm plate.
  e. After 20 min incubation, add the DNA-Lipofectamine 2000 mixture dropwise to cells and agitate to distribute evenly.
  f. After 5–6 h, change the medium and replace with 10 mL fresh culture medium for a 10 cm plate.
4. Start to collect the viral supernatant after 72 h.
  a. Obtain a 15 mL tube and label the tube with “VirusName_Sup1”.
  b. Collect all supernatant (total around 8–9 mL due to evaporation) into the tube carefully.
  c. Replace the medium with 7 mL culture medium carefully as soon as possible.
    ***Note:*** Set the flow rate of the pipette to the minimum and slowly blow the medium along the plate edge into the culture plate to avoid disrupting the attached cells.
  d. Spin supernatant at 2000 rpm (724 g) for 5 min to pellet cell debris.
  e. Filter the supernatant through a 0.45 μm membrane filter.
  f. Retain 200 μL of the supernatant for virus titering assay.
  g. Store the supernatant and the aliquot at −20°C for short time storage.
    ***Note:*** The 200 μL aliquot should be stored at −20°C too. The aliquot is for avoiding from repeated freeze-thaw cycles of the supernatants. This is only for short time storage; you should concentrate the supernatants as soon as possible. For long time storage, the supernatants should be stored at −80°C.
5. Collect the supernatant daily for 5 days, and process these supernatants each day in the same way described in step 4.

***Note:*** For rescued rabies virus in this protocol, you have 6 supernatants in total. For CVS-N2cΔG rabies virus, you need collect the supernatants every two days until Day-13 to collect 6 supernatants in total.

**Table 1:** Summary of different rescue conditions for four different recombinant rabies virus used in this protocol

Rabies virus name	Cell lines used for virus rescue	Plasmid: Transfection reagent ratio	Cell lines used for virus passage	Cell lines used for titering using FACS
RVΔG-4mCherry (B19G)^3^	HEK-293T	1:2 PEI	BHK-B19G-2	HEK-293T
RVΔG- 4EGFP (B19G)	HEK-293T	1:1.18 Lipofectamine 2000	BHK-B19G-2	HEK-293T
CVS-N2cΔG-mCherry (B19G)^2^	HEK-293T	1:1.18 Lipofectamine 2000	HEK-293T-B19G-1-1	HEK-293T
RVΔL-5Cre^1^	HEK-293T	1:1.18 Lipofectamine 2000	BHK-B19L-1-2	HEK-293T-FLEX- BC-1 (Cre recombinase dependent mCherry reporter cell line)
RVΔG- 4mCherry(EnvA)	–	–	BHK-EnvA	HEK-293T-TVA

**Table 2:** List of the amount of each plasmid for a 10 cm plate RVΔG rabies virus rescue

Plasmid	Amount/10 cm plate (μg)
pRVΔG genomic plasmid	24.5
pCAG-B19N	12.4
pCAG-B19P	6.6
pCAG-B19G	4.8
pCAG-B19L	5.5
pCAG-T7pol	7.4

**Table 3:** List of the amount of each plasmid for a 10 cm plate CVS-N2cΔG rabies virus rescue

Plasmid	Amount/10 cm plate (μg)
pCVS-N2cΔG genomic plasmid	24.5
pCAG-N2c-N	12.4
pCAG-N2c-P	6.6
pCAG-B19G∗	4.8
pCAG-N2c-L	5.5
pCAG-T7pol	7.4

∗ Note: For rescue the N2c strain, using B19G is better than using N2cG.

### Preparation two: Rabies virus passaging, purification, and pseudotyping

**Timing: 3 weeks**

This section describes the steps for rabies virus passaging, purification and pseudotyping. Before start virus passaging, you need to titrate each supernatant of rescued rabies virus. After titration, choose several higher titer supernatants for passaging.

### Titration of rescued rabies virus supernatants

6. Prepare HEK-293T in 96-well plate (approximately 30,000 cells/well) for rescued virus supernatants titration.

***Note:*** For 6 supernatants described in previous steps, prepare 3 dilutions for each one. Thus, in this step prepare 6 supernatants ∗3 dilutions + 1 counting-well + 1 negative-control = 20 wells of cells. The one additional well of cells is for the cell counting prior to adding the virus supernatant.

7. Prepare 3 dilution series in 1.5 mL tube for each of the rescued rabies virus supernatants.

**Table 4:** Dilution scheme of rescued virus supernatants for FACS titration

Dilution series	Volume of culture medium (μL)	Volume of supernatant/previous dilution (μL)
Dil.0	297	33
Dil.1	297	33
Dil.2	297	33

***Note:*** For 6 supernatants, this would mean that you have 18 diluted supernatants in total to be tested. Table 4 (see materials and equipment setup part) lists the dilution series for each supernatant.

8. Digest the cells down.
  a. Aspirate medium from only one well.
  b. Add 100 μL DPBS (1X) each well to wash cell.
  c. Discard the DPBS.
  d. Add 50 μL 0.25% trypsin.
  e. Incubate for 2 min.
  f. Add 100 μL culture medium, and triturate until uniformly resuspended.
  g. Count the cell number using hemocytometer.

**CRITICAL:** This cell counting step is important for subsequent calculation of virus titer.

9. Design the samples that need to be added to each well of the 96-well plate and label sample names in the plate.
10. Aspirate medium from the wells gently.
11. Adjust the pipette to 200 μL, and add the diluted supernatants into the wells of the 96-well plate.
12. After 3 days, inspect titer wells under fluorescence microscope to have an initial assessment regarding cell health.
13. Prepare for FACS.
  a. Digest the cells down as step 8.
  b. Add 50 μL 4% PFA to each well.
  c. Wrap up the 96-well plate with aluminum foil to protect from light.
  d. Analyze the percentage of fluorescent cells by flow cytometry.
14. Calculate the virus titer.
  a. Calculate multiplicity of infection with MOI equation^4^ with the percentage of fluorescent cells around 0.2%–8%(P).
    MOI=−Ln(1−P)
    ***Note:*** P refers to the percentage of fluorescent cells which means the infected cells.
  b. Calculate the viral infection units with equation: Viral infection units = MOI∗ Cell numbers that uses for tittering.
    ***Note:*** For example, for one Dil.2 well with 6.6E4 cells, the percentage of fluorescent cells is 3.1%, then we can get the virus infection units in this well= (-Ln(1–3.1%)) ∗ 6.6e4 = 2078, the titer of this supernatant should be 2078 IU/0.02 mL (virus volume)∗100 (dilution factor) = 1.039∗E7 IU/mL.

### Recombinant rabies virus passaging

15. Prepare two 15 cm plates of cells (2E6 to 3E6 cells per plate) for rabies virus passaging. Incubate for 18–24 h.
16. Digest the cells down.
  a. Aspirate medium from only one plate.
  b. Add 5 mL DPBS(1X) each well to wash cell.
  c. Discard the DPBS.
  d. Add 4 mL 0.25% trypsin.
  e. Incubate for 2 min.
  f. Add 8 mL culture medium.
  g. Triturate until uniformly resuspended.
17. Count the cell number using hemocytometer.
18. Inoculate another plate of cells with the rescued rabies virus supernatant.

**CRITICAL:** The titer of supernatants used for passaging need to be more than E5 IU/mL. The MOI need to be 0.1. Calculate the volume of rescued rabies virus supernatant need to be added according to the virus titer determined by previous FACS titration steps. The volume of supernatants added need to be less than 10 mL. Add the medium until the total volume reaches 15 mL.

19. Start to collect the viral supernatant after 24 h.
  a. Obtain a 50 mL tube and label the tube with “VirusName_P1_Sup1.”
  b. Collect 15 mL supernatant into the tube carefully.
  c. Replace the medium with 15 mL culture medium carefully as soon as possible.
  d. Spin the supernatant at 2000 rpm (724 g) for 5 min.
  e. Filter the supernatant through 0.45 μm membrane filter and store it at −20°C.
  f. Retain 200 μL of the supernatant for virus tittering assay and store at −20°C.
20. Collect the supernatants from the Day-2 to Day-9 and process them in the same way described in step 19.
21. Determine the titer of each supernatant using FACS as described in step 6-14.

### Concentration and purification of passaged RV

22. Leave the supernatants in 4°C for 18–24 h to thaw thoroughly. Then pool all supernatants in a 150 mL bottle.
23. On the following day, place the filtered supernatants at ∼25°C for at least 1 h.
24. Incubate the supernatants with 30 U/mL Benzonase at 37°C for 1 h.
25. Prepare for the spins.
  a. Load 33 mL of supernatants into 4 conical-bottom ultra-centrifuge tubes,
  b. Underlay the supernatants with 5 mL 25% sucrose in DPBS (1X) with a long cannula with a 10 mL syringe.

***Note:*** Prepare 25% sucrose just prior to its use. Dissolve 6 grams of sucrose in DPBS (1X) to make a final volume of 24 mL at 37°C. Let it stand for one hour, and then filter it through a 0.22 μm membrane filter before using it.

26. Switch on the ultracentrifuge and set the temperature to 4°C
27. Balance the buckets and then spin at 22000 rpm (82700 x g) using SW 32 Ti for 2 h at 4°C.

***Note:*** After the spins, a pellet will be visible. If the amount of virus is too low, you may not see a pellet.

28. Quickly turn the tubes upside down to discard the supernatants into the waste receptacle.
29. Suck up all the remaining droplet.
30. Resuspend each tube with 18 μL cold DPBS (1X).
31. Put the centrifuge tubes back into the buckets and close the lid.
32. Place them on a 50 mL tube rack and shake in the cold room for 18–24 h on a flat shaker.
33. Transfer the liquids of the four tubes into a single 1.5 mL tube and label it, and place it on ice.
34. Decontaminate with 75% ethanol and discard the four centrifuge tubes.
35. Clean the buckets, conical adaptors and rack.
36. Aliquot the concentrated virus.
  a. Put an appropriate number of cryogenic tubes on the cold tube rack in the bio-safety cabinet.
  b. Loose the caps to allow for later removal with one hand.
  c. Gently mix the virus suspension by gently pipetting up and down several times.
  d. Spin at 1000 rpm for 1 min to dissolve the froth.
  e. Aliquot the virus 5 μL per tube.
  f. Label the tubes with “VirusName-P1-Date”.
  g. Freeze them at −80°C as soon as possible.
37. Titrate for concentrated virus using FACS.

**Table 5:** Dilution scheme of concentrated virus for FACS titration

Dilution series	Volume of culture medium (μL)	Volume of virus/previous dilution (μL)
Dil.0	330	0.825
Dil.1	297	33
Dil.2	297	33
Dil.3	297	33
Dil.4	297	33

***Note:*** Prepare dilution series of concentrated virus following steps 6-14 except for the volume of virus added in Dil.0 at step 7. The dilution series are described in Table 5 (see materials and equipment setup part).

### Pseudotyping rabies virus with EnvA glycoprotein

38. Prepare two 15 cm plates of BHK-EnvA^5^ cells for rabies virus passaging.

***Note:*** The cell number of each plate is in the range of 2E6 to 3E6 cells.

39. Incubate for 18–24 h.
40. Count the cell number using one of the plates as described in step 16-17.
41. Inoculate another plate of cells with the concentrated rabies virus.

**CRITICAL:** The MOI need to be 2. The preparation of virus supernatants is same as step 18 except for the MOI need to be reached.

42. Wash away the B19G coated virus residue.
  a. Discard the supernatant after 24 h.
  b. Wash cells with 10 mL DPBS (1X) twice.
  c. Add 15 mL medium.
  d. Repeat steps above after another 24 h.
43. Collect the supernatants from the Day-3 to Day-5 and process them in the same way described in step-19.
44. Determine the titer of each supernatant using FACS as described in step 6-14.

**CRITICAL:** HEK-293T-TVA cell line need to be used for titration of EnvA-coated rabies virus.

## Key resources table

**Table:** 

REAGENT or RESOURCE	SOURCE	IDENTIFIER
**Chemicals, peptides, and recombinant proteins**		

Poly-L-lysine 0.01%	Sigma-Aldrich	CAT #: P4832-50ml
DPBS	Gibco	CAT #: 14190144
0.05% trypsin-EDTA (1X)	Gibco	CAT #: 25300054
DMEM	Gibco	CAT #: C11995500BT
Fetal bovine serum	Sigma-Aldrich	CAT #: F0193-500ML
Antibiotic-antimycotic (100X)	Gibco	CAT #: 15240062
Opti-MEM medium	Gibco	CAT #: 11058021
Lipofectamine 2000 reagent	Invitrogen	CAT #: 11668019
4% PFA	Leagene	CAT #: DF0135/500ml
Benzonase	Haigene	CAT #: C2002-100KU
Sucrose	Sigma-Aldrich	CAT #: V900116-500G
PEI, polyethylenimine linear	BIOHUB	CAT #: 78PEI40000-1g
EndoFree mini plasmid kit	TIANGEN	CAT #: DP118-02
DNase I, recombinant DNase I (RNase-free)	TaKaRa	CAT #: 2270A

**Critical commercial assays**		

TaKaRa MiniBEST viral RNA/DNA extraction kit v.5.0	TaKaRa	9766
PrimeScript II 1st strand cDNA synthesis kit	TaKaRa	6210A
PowerUp SYBR green master mix for qPCR	Applied Biosystems	A25742

**Software and algorithms**		

QuantStudio real-time PCR software v.1.3	Applied Biosystems	https://downloads.thermofisher.com/Analysis_Software/qPCR/QuantStudio_Real-Time_PCR_Software/1.3/QuantStudio_Real_Time_PCR_Software_v1.3.zip
LightCycler 96 SW 1.1	Roche	https://lifescience.roche.com/global/en/products/product-category/lightcycler.html#4

**Oligonucleotides**		

Target	forward primer	reverse primer
RV P-M	GTGTACTGGGATGGGTCGCT	CCTCGTCCCTGCGGTTTTT
RVG	GTTCACTTGCACAGGCGTTG	CCAGTTGTACGCGGCTCTAC
RVG for cell line	ACCAGAATCGAAACAACGCAG	TGATATCGAATTCCTGCAGCC
RVG for virus package	ATCAGAACCTACGCAACACAATC	GCATTGGCCACACCAGCC
RVL	GCCAACTACATCTTGCCAC	ACACTCTCTTCAATCCAAACAC
CVS-N2c N-P	CCAATCATCAAGCCCGTCCA	GCCATCTCAAGATCGGCCAG
CVS-N2c G	CAACCTGTCCGAGTTCTCCT	CTTCCAGTTATACGCGGCTCT
RV RT primer	**GCTTTAAGGCCGGTCCTAGCAA** CAAGCCCGTCCAAACTCATTC	Note: Sequence in bold is added CCS1 sequence which is optional.
CVS-N2c RT primer	CCAATCATCAAGCCCGTCCA	–

**Other**		

FACSAria sorter	BD Biosciences	BD FACSAria III
LightCycler 96 instrument	Roche	CAT#: 05815916001
Qubit 4 fluorometer	Thermo Fisher Scientific	CAT#: Q33238
Research slide scanner	EVIDENT	SLIDEVIEW VS200
1.5 mL tube	BIOFIL	CAT #: CFT003015
10 mL syringe	BIOFIL	CAT #: GSP110010
Ultracentrifuge	Beckman Coulter	CAT # Optima XE-90
SW 32 Ti	Beckman Coulter	Product No:369694
Long cannula	Taobao	N/A
38.5 mL, Open-Top Thinwall Polypropylene Centrifuge Tube	Beckman Coulter	CAT #: 326823
0.45 μm Stericup filter	Millipore	CAT #: SE1M003M00
PCR 8 (EU 8-tube strip)	GeneBrick	CAT #: GP020813
Hemacytometer	Tansoole	CAT #: 2030724
MicroAmp fast optical 96-well reaction plate	Thermo Fisher Scientific	CAT #: 4306737
MicroAmp 96-well optical adhesive film	Thermo Fisher Scientific	CAT #: 4360954
E4 multi pipette E8-300XLS+	RAININ	CAT #: E8-300XLS+
TGear plate centrifuge	TIANGEN	CAT #: OSE-MP25
Digital stereotaxic instruments	RWD	CAT #: 68018
Auto-nanoliter injector	Drummond	CAT #: Nanoject II
Glass capillaries	Drummond	CAT #: 3-000-203-G/X
Isoflurane	RWD	CAT #: R510-22-10
96-well plate	BIOFIL	CAT #: TCP011096
10 cm plate	BIOFIL	CAT #: TCD010100
15 cm plate	BIOFIL	CAT #: TCD110150

## Materials and equipment

Materials and equipment are found in the following tables.

## Step-by-step method details

### Part 1: Preparation of standards for RT-qPCR

**Timing: 3 days**

This section describes methods to prepare plasmid stocks utilized for generating standard curves. Here we use rabies virus genomic plasmids as standards to generate accurate copy number of rabies virus. For RVΔG viruses we use pRVΔG-4mCherry plasmid as standards. As the qPCR primers are designed on the common region, no matter what the gene was inserted at Glycoprotein(G) gene site, we can use the same standards.

1. Transform the plasmid that is utilized for drawing the standard curve.

***Note:*** To avoid cross-contamination, do not prepare different plasmids at the same day.

2. Culture the colony from step-1 and extract the plasmid from E.coli following plasmid extraction kit protocol.

***Note:*** To maintain the plasmid as stable as possible, TE buffer should be used for the elution and dilution.

3. Measure plasmid concentration using NanoDrop spectrophotometer.
4. Dilute the plasmid to 20–50 ng/μL with TE buffer.
5. Determine the final concentration with the average of three trials using NanoDrop spectrophotometer.

***Note:*** The concentration measured by NanoDrop or Qubit 4 Fluorometer is similar.

6. Aliquot the standards.
  a. Aliquot parts of the standards into 10 μL per 0.2 mL PCR tube.
  b. Store aliquot for the confirmed standards at −20°C.

**Table 6:** Standards for three different recombinant rabies virus

Plasmid name	Length	Concentration(ng/μL)	Copy number after 10x dilution in 1 μL
pRVΔG-4mcherry	14497 bp	32.03	2.160e+8
pRVΔL-5Cre	10006 bp	30.83	3.012e+8
pN2cΔG-mcherry	14919 bp	68.867	4.498e+8

**Pause point:** Store the rest at – 80°C for longer reservation.

***Note:*** Sequencing the plasmid to check if it has the correct sequence. Calculate copy number with NEBiocalculator. The standards used in our experiment are listed in Table 6 (see materials and equipment setup part). For example, for the pRVΔG-4mcherry plasmid standards, the concentration used for storage is 32.03 ng/μL, but the first dilution of standards used for standard curve depicting is 10x dilution of the storage. It means we use 3.203 ng pRVΔG-4mcherry plasmid DNA as the first standards, the copy number correspondence with 3.203 ng pRVΔG-4mcherry plasmid DNA is 2.160e+8 calculated by NEBiocalculator.

**CRITICAL:** Keep noting the Ct value of each standard along with time. They ought to stay within 0.5 Ct of their initial value. Once the Ct value of the standard begins to drift, it is the time to create a new one or take out the rest plasmids from −80°C to make new aliquots.

### Part 2: Rabies virus genome RNA extraction

**Timing: 2 h**

This section describes the steps to extract virus genomic RNA from purified rabies virus using TaKaRa MiniBEST Viral RNA/DNA Extraction Kit. Other viral RNA/DNA extraction kits can also be used. Using a space designated for RNA-based experiment is recommended. Clean the bench with RNase decontamination solution before you start these following steps. Appropriate personal protective equipment needs to be used during all the process.

7. Lysis of virus.
  a. Obtain one aliquot of purified rabies virus from −80°C refrigerator.
    i. Thaw on ice.
    ii. Supplement purified virus (5 μL in our case) with RNase free dH2O to 200 μL volume and transfer to a 1.5 mL tube.
  b. Add 200 μL Buffer VGB, 20 μL Proteinase K and 1 μL Carrier RNA, and mix them thoroughly.
  c. Incubate at 56°C for 10 min (dry bath preferred).
8. Add 200 μL of 96–100% ethanol into the lysate and mix thoroughly.
9. Place the Spin Column in the collection tube.
  a. Move the solution to the spin column.
  b. Centrifuge at 12,000 rpm for 2 min.
  c. Discard the filtrate.
10. Wash and dry the silica membrane.
  a. Add 500 μL Buffer RWA into the Spin Column.
  b. Centrifuge at 12,000 rpm for 1 min.
  c. Discard the filtrate.
  d. Add 700 μL Buffer RWB into the Spin Column
  e. Centrifuge at 12,000 rpm for 1 min.
  f. Discard the filtrate.
  g. Repeat step d to step f.

***Note:*** Please make sure that a specified volume of 96–100% ethanol has been added to Buffer RWB. Add Buffer RWB around the Spin Column wall to help completely wash the salt attached to the wall.

11. Place the Spin Column on the Collection Tube and centrifuge it at 12,000 rpm for another 2 min.
12. Put the Spin Column on a new 1.5 mL RNase-free collection tube and add 32 μL RNase-free dH2O to the center of the Spin Column membrane. Stand at room temperature for 5 min.
13. The RNA is eluted by centrifuging at 12,000 rpm for 2 min.
14. Prepare DNase I treatment mixture as described in Table 7 in a 0.2 mL PCR tube.
  ***Note:*** Store the rest of the RNA in −80°C as soon as possible.
  ***Optional:*** Ethanol precipitation of RNA.
  a. Supplement the above solution to 100 μL with RNase-free water.
  b. Add 10 μL 3M sodium acetate and 250 μL cold ethanol.
    ***Note:*** Pre-cool the ethanol at −20°C.
  c. Mix and place at −80°C for more than 20 min.
  d. Centrifuge at 15,000 rpm at 4°C for 25 min.
    ***Note:*** A white pellet is visible.
  e. Discard the supernatant.
  f. Add 70% cold ethanol to wash the pellet.
    ***Note:*** Pre-cool the 70% ethanol at −20°C.
  g. Centrifuge at 12,000 rpm at 4°C for 5 min
  h. Discard the supernatant.
  i. Dry the precipitation for about 10 min.
  j. Add 16 μL of water to dissolve the RNA.
    ***Note:*** To be consistent with the initial RNA amount for subsequently easy calculation we dissolve the RNA into 16 μL of water.
    **CRITICAL:** Handle the fresh extracted RNA as soon as possible. If the subsequent steps can’t be initiated immediately, store the RNA samples in the −80°C, not exceeding one week.

**Table 7:** DNase I treatment reaction setup

DNase I treatment	Volume per reaction (μL)	Stock concentration	Final concentration
RNA	16	–	–
Recombinant DNase I (RNase-free)	2	5 U/μL	0.2 U/μL
RNase Inhibitor	0.5	40 U/μL	0.4 U/μL
DNase buffer	5	10x	1x
RNase free dH2O	26.5	–	–

### Part 3: Reverse transcription of rabies virus RNA

**Timing: 2.5 h**

This section describes the procedure for reverse transcribe the rabies virus RNA into cDNA, following the instruction of PrimeScript II 1st Strand cDNA Synthesis Kit. The application of other reverse transcriptase is possible as well. The RT primer (ref to key resources table) is designed against the negative rabies virus genome RNA.

15. Prepare the RT1 reaction mixture as Table 8 described in a 0.2 mL PCR tube.

**Table 8:** RT1 reaction setup

RT1	Volume per reaction(μL)	Stock concentration	Final concentration
RT-primer	1	50 μM	5 μM
dNTP Mixture	1	10 mM each	1 mM each
RNA	8	–	–
16. Keep warm at 65°C for 5 min, then quickly cool on ice.

***Note:*** The above processing can denature the template RNA and increase the reverse transcription efficiency.

17. Prepare RT2 mixture as Table 9 described in the above-mentioned 0.2 mL PCR tube, with a total amount of 20 μL.

**Table 9:** RT2 reaction setup

RT2	Volume per reaction(μL)	Stock concentration	Final concentration
RT1 solution	10	–	–
PrimeScript II Buffer	4	5×	1x
RNase Inhibitor	0.5	40 U/μL	1 U/μL
PrimeScript II RTase	1	200 U/μL	10 U/μL
RNase Free dH2O	Up to 20 μL	–	–
18. Mix gently and centrifuge to the bottom of the tube.
19. Incubate the reverse transcription reaction at 50°C for 1 h, 70°C for 15 min.

**CRITICAL:** The deactivation process is carried out at 70°C for 15 min, other than 95 min for 5 min, to protect the long rabies virus genome from being broken.

20. Put the reaction tube on ice or at 4°C immediately after reverse transcription.

***Note:*** Process the sample as soon as possible, and if the subsequent steps can’t be started immediately, the reaction solution needs to be placed at −20°C.

### Part 4: RT-qPCR for rabies virus titration

**Timing: 4 h**

This section outlines the RT-qPCR steps and data analysis methods for titrating the copy number of rabies virus.

21. Prepare the standards dilution series.
  a. Take out the aliquot of standards from step 6 from −20°C refrigerator.
  b. Thaw on ice.
  c. Obtain six 0.2 mL PCR tubes and label them with “STD.1-6”.
  d. Place the tubes on ice.
  e. To the tube labeled “STD.1”, add 49 μL of nuclease free water.
  f. To the other 5 tubes, add 45 μL of nuclease free water.
  g. Add 1 μL Standard stock to the tube labeled “STD.1”, vortex thoroughly.
    ***Note:*** When preparing each diluted sample, keep the lids of the other tubes closed to prevent contamination.
  h. To the tube labeled “STD.2” add 5 μL of “STD.1”, vortex thoroughly and spin down.
    ***Note:*** The dilution process for the rest tubes is depicted in the Table 10.
    **CRITICAL:** Every STD sample must be mixed thoroughly before taking 5 μL for the preparation of the next dilution of the sample. If the mixing is not sufficient, high-quality standard curves can’t be obtained finally. Low copy number samples degrade quickly, therefore dilution of the standards must be done on the same day as the RT-qPCR, in order to report the accurate copy number. DON’T keep the standard dilute solutions.

**Table 10:** Dilution scheme for standards applied in qPCR reaction

Dilution series	Volume of sample(μL)	Nuclease free dH2O(μL)	Dilution factor	Total dilution
STD.1	1 μL Standard stock	49	50x	10x∗
STD.2	5 μL STD.1	45	10x	100x
STD.3	5 μL STD.2	45	10x	1000x
STD.4	5 μL STD.3	45	10x	10000x
STD.5	5 μL STD.4	45	10x	100000x
STD.6	5 μL STD.5	45	10x	1000000x

∗ Note: Since we add 5 μL STD sample during qPCR reaction setup (refer to step 22, Table 12), the total dilution of STD.1 from the original STD sample should divide by 5 (1 μL Standard stock/50∗5 = 0.1 μL).
22. Prepare virus RNA RT reaction solution dilution series.
  a. Keep the RT2 solution on ice during the process.
  b. Obtain three 0.2 mL PCR tubes and label them with “SampleDil.1-3”. Place the tubes on ice.
  c. To the tube labeled “SampleDil.1” add 40 μL of nuclease free water.
  d. To the rest 2 tubes add 45 μL of nuclease free water.
  e. Add 10 μL RT2 solution to the tube labeled “SampleDil.1”, vortex thoroughly.
  f. To the tube labeled “SampleDil.2” add 5 μL of “SampleDil.1”, vortex thoroughly.
  g. To the tube labeled “SampleDil.3” add 5 μL of “SampleDil.2”, vortex thoroughly. The dilution process is described in Table 11 (see materials and equipment setup part).

**Table 11:** Dilution scheme for cDNA products used in qPCR reaction

Dilution series	Volume of sample(μL)	Nuclease free dH2O(μL)	Dilution factor
SampleDil.1	10 μL cDNA product	40	5x
SampleDil.2	5 μL SampleDil.1	45	10x
SampleDil.3	5 μL SampleDil.2	45	10x

**CRITICAL:** Every sample dilution must be mixed thoroughly before taking 5 μL for the preparation of the next dilution of the sample to obtain accurate copy number of the virus.

23. Prepare rabies virus titration RT-qPCR master mix.
  a. Make a master mix of each pair of primers (ref to key resources table) in a 1.5 mL tube without adding RT2 samples or standards as Table 12 described.
    ***Note:*** For each rabies virus RT-qPCR sample, prepare 30 reactions with 10% surplus for each pair or primers.

**Table 12:** RT-qPCR master mix reaction system

RT-qPCR master mix	Volume per reaction(μL)	Stock concentration	Final concentration	Volume per rabies virus RT-qPCR reaction (μL) x33
powerup SYBR mix	10	2x	1x	330
Forward primer	1	10 μM	0.5 μM	33
Reverse primer	1	10 μM	0.5 μM	33
RNase Free dH2O	3	–	–	99
  b. Pipette the master mix up and down for 15 times.
    ***Note:*** Carefully avoid bubbles.
24. Set up the rabies virus RT-qPCR plate.
  a. Obtain a 96-well reaction plate and place on ice.
  b. For master mix for each pair of primers pipette 15 μL of master mix into 30 reaction wells of the 96- well reaction plate on ice.
  c. Add 5 μL diluted series of STDs or SampleDils previously prepared into each well using multichannel pipette.
    ***Note:*** The qPCR plate setup example is shown in Table 13. Before you start to add the samples and standards, organize them appropriately for convenience.

**Table 13:** Example of qPCR plate setup

STD.1	STD.1	STD.1	G-STD.1	G-STD.1	G-STD.1	SampleDil.1	SampleDil.1	SampleDil.1
STD.2	STD.2	STD.2	G-STD.2	G-STD.2	G-STD.2	SampleDil.2	SampleDil.2	SampleDil.2
STD.3	STD.3	STD.3	G-STD.3	G-STD.3	G-STD.3	SampleDil.3	SampleDil.3	SampleDil.3
STD.4	STD.4	STD.4	G-STD.4	G-STD.4	G-STD.4	G-SampleDil.1	G-SampleDil.1	G-SampleDil.1
STD.5	STD.5	STD.5	G-STD.5	G-STD.5	G-STD.5	G-SampleDil.2	G-SampleDil.2	G-SampleDil.2
STD.6	STD.6	STD.6	G-STD.6	G-STD.6	G-STD.6	G-SampleDil.3	G-SampleDil.3	G-SampleDil.3
Blank	Blank	Blank	G-Blank	G-Blank	G-Blank	–	–	–
  d. Adhere the 96-well optical adhesive film onto the plate carefully.
  e. Centrifuge at 2800 rpm for 15 s to centrifuge the reaction liquid and to mix them evenly using TGear Plate Centrifuge.
25. Set up the qPCR program as Table 14 described.

**Table 14:** Setup of qPCR program

Temperature	Rate of temperature increase	Time	Cycle
50°C	1.6 °C/s	2 min	1x
95°C	1.6 °C/s	10 min	
95°C	1.6 °C/s	15 s	40x
60°C	−1.6 °C/s	1 min	

Note: When the Ct value seems not reliable you can add a melting curve program to see if there are unspecific PCR products during this qPCR program.
26. Start the program.
27. Calculate rabies virus titer through RT-qPCR results.
  a. Draw the standard curve by utilizing the Ct value versus copy number of the STDs.
  b. Apply logarithmic fitting to get the standard curve formula and the R2 value.
    ***Note:*** The R2 value ought to be nearly one.
  c. Put the sample’s Ct value into the formula from sub-step b to calculate the corresponding copy number.
  d. Calculate the dilution ratio of the sample relative to the initial virus sample.
  e. Multiply the copy number by the dilution ratio to calculate the titer of the virus.
  f. Use the average titer of the three sample dilution series as the final titer of the rabies virus.
    **CRITICAL:** Make sure that the copy number of the sample is within the range of the standard curve.
    ***Note:*** In the Expected Outcomes parts, we offer an example to demonstrate the calculation process. You can re-calculate the example data to get a better understanding.

### Part 5: Stereotaxic rabies virus injection for titer verification

**Timing: 2 weeks**

This section introduces the approach of verification of rabies virus titer on mouse through stereotaxic injection.

**Figure 1:**
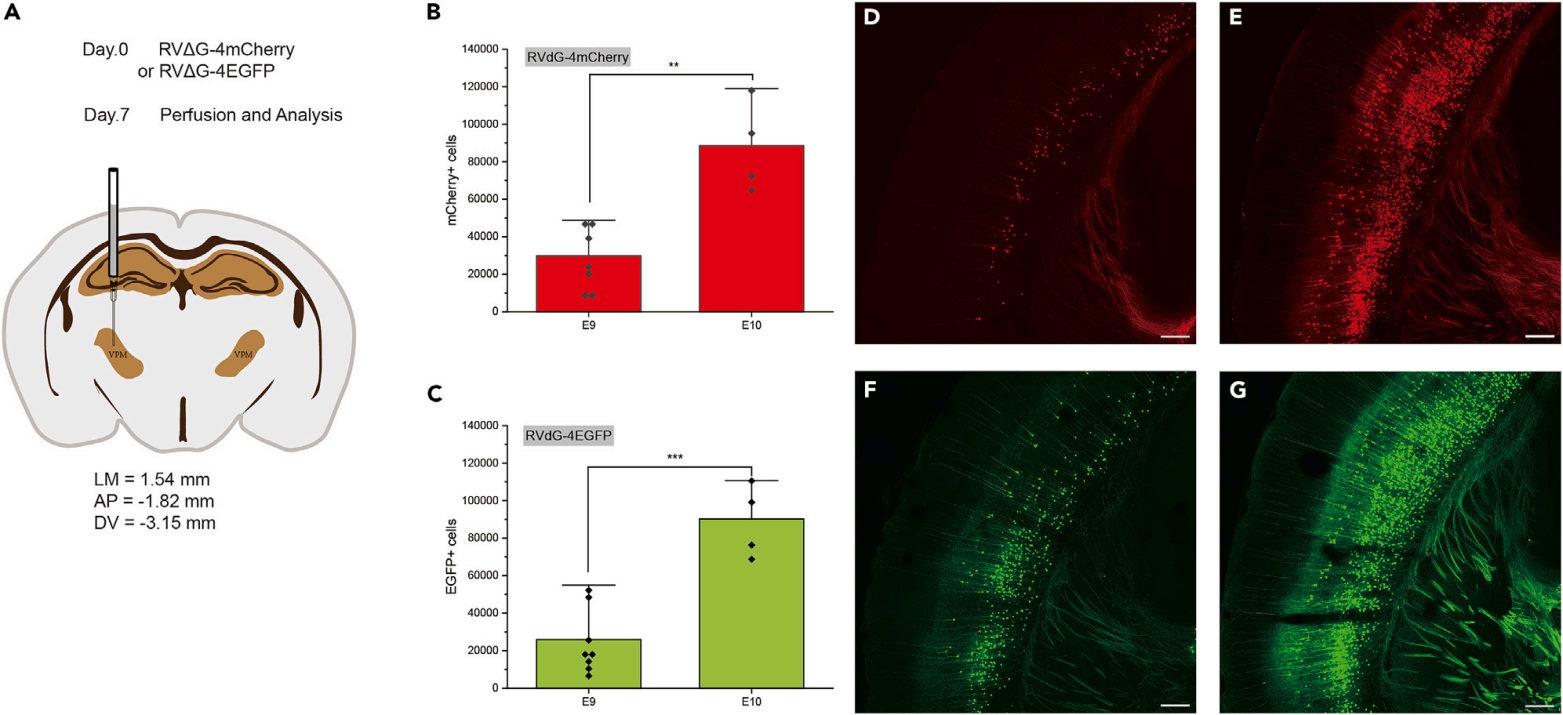
Verification of rabies virus titer through stereotaxic injection (A) Schematic diagram of stereotaxic injection brain region and the coordinates. (B–C) Statistical analysis of different number of labeled neurons after injection of RVΔG-4mCherry or RVΔG-4EGFP with titer of E9 or E10. Data are represented as mean ± outlier. Each dot represents six times of the total number of one series consisting of every sixth 50 μm section from an individual mouse brain. Unpaired two-sided Student’s t test was performed for comparison, ∗∗p < 0.01, ∗∗∗p < 0.001. (D–F) Representative pictures of different number of labeled neurons after injection of RVΔG-4mCherry or RVΔG-4EGFP with titer of E9 or E10. Scale bars: 200 μm.

***Note:*** The specific steps of stereotaxic injection has been described.^6^ The coordinates used in this protocol is anteroposterior (AP) = −1.82 mm with respect to (w.r.t.) bregma, lateromedial (LM) = 1.54 mm w.r.t bregma, dorsoventral (DV) = −3.15 mm w.r.t the brain surface (Figure 1). The mouse stereotaxic rabies virus injection experiments are listed in Table 15 (see materials and equipment setup part). The labeling efficiency of rabies virus with E10 titer is significantly higher than that of rabies virus with E9 titer (Figure 1B–1G and supplementary file 1-3), which offer evidence for the reliability of titration through qPCR and FACS.

***Optional:*** Rabies virus infected neuronal cell counting.

28. Count neuronal cell number infected by rabies virus.
  a. Open ImageJ software,^7^ select File → Open command to open the TIF image file.
  b. Use Image → type → 16-bit command to convert the file type.
  c. Select the fourth icon below the image to circle the entire fluorescence cortex region.
  d. Use Image → Crop command to crop the ROI.
  e. Use Image → Adjust → Threshold command to adjust the threshold to 500-3296 points.
  f. Use Process → Binary → Watershed command to apply cell segmentation.
  g. Use Analyze → Analyze Particles cell counting. Set the Size (inch) to 0.01-800 and the Circularity: 0.2-1.0. Tick the Clear results and Summarize options.
  h. Record the Count value as the cell number labeled with the injected virus.

**Table 15:** List of mouse stereotaxic rabies virus injection experiments

Virus	Titer by FACS (IU/mL)	Volume	Mouse Quantity	Mouse number
RVΔG-4mcherry (B19G) E10	4.43E+10	200 nL	4	E10 E12 E13 E14
RVΔG-4mcherry (B19G) E9	4.43E+09	200 nL	8	E15 E16 E17 E18 E46 E47 E48 E49
RVΔG-4EGFP (B19G) E10	4.49E+10	200 nL	4	E22 E23 E24 E25
RVΔG-4EGFP (B19G) E9	4.49E+09	200 nL	8	E26 E27 E28 E29 E42 E43 E44 E45
RVΔL-5Cre E10	5.37E+10	250 nL	4	A5 A10 A54 A59

## Expected outcomes

Here we present an example of a 5 μL RVΔG-4EGFP rabies virus sample. The average titer measured by three time of repeated FACS is 6.39E10 IU/mL. After RNA extraction, we obtained total 275.2 ng RNA (8.6 ng/μL in 32 μL RNase-free water). We then performed reverse transcription reaction following described steps resulting in 20 μL RT product.

**Figure 2:**
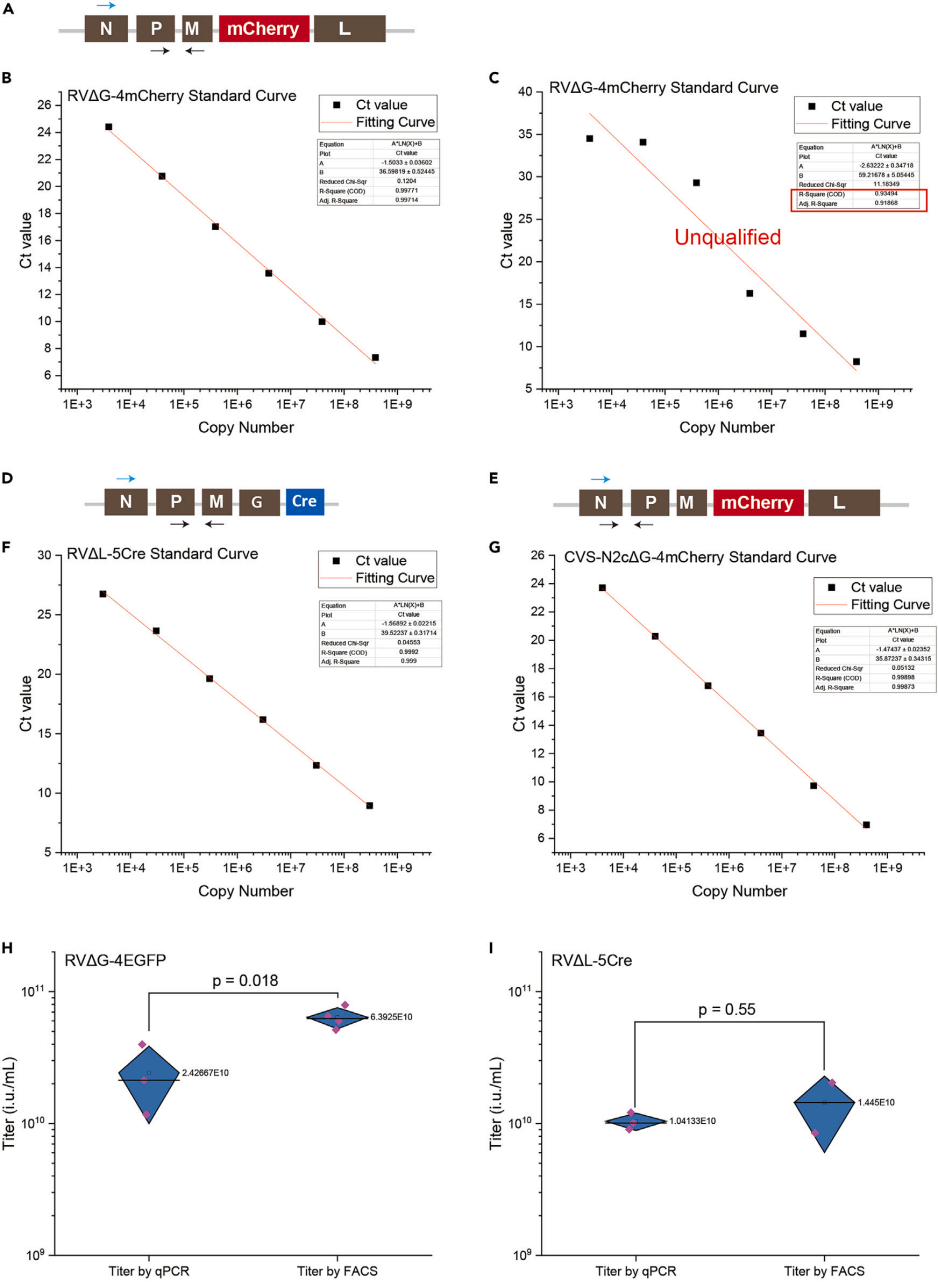
Example of standard curves and comparison of titration between FACS and RT-qPCR (A) Schematic diagram of RVΔG-4mCherry rabies virus genome indicated with the positions of the reverse transcription primers (blue arrow) and qPCR primers (black arrows). (B) RVΔG-4mCherry standard curve. The square dots are Ct values measured by qPCR experiment. The red line depicted the filling standard curve. Equation Y = A∗ln(X)+B is used for fitting. The R-Square(R2) is 0.99771(nearly 1). (C) RVΔG- 4mCherry standard curve that is unqualified with the R2 far from one. (D)Schematic diagram of RVΔL- 5Cre rabies virus genome indicated with the positions of the reverse transcription primers (blue arrow) and qPCR primers (black arrows). (F) RVΔL-4mCherry standard curve. (E) Schematic diagram of RVΔL- 5Cre rabies virus genome indicated with the positions of the reverse transcription primers (blue arrow) and qPCR primers (black arrows). (G) RVΔL-4mCherry standard curve. (H and I) Comparison of RVΔG-4EGFP and RVΔL-5Cre rabies virus measured by qPCR and FACS. Data are represented as mean ± SD. Each dot represents an individual titer value. Unpaired two-sided Student’s t test was performed for comparison, as indicated.

For the quantitative PCR reaction plate containing standards and controls, we used the plate layout described in Table 13 (see materials and equipment setup part). In Table 16 (see materials and equipment setup part) we present the example Ct value for each of the sample one to one correspondence with Table 13. The RT primer and P-M primer pair position is shown in Figure 2A. The results for G primer pair are listed as G-STD.x or G-SampleDil.x and others are the results for P-M primer pair. It is shown in Figure 2B of an example standard curve and the standard curve equation for RVΔG. Ct values for G primer pair show that there’s no contamination in this virus sample.

**Table 16:** Example of qPCR reaction Ct value results

10.22	10.17	9.91	–	–	–	18.64	18.58	18.85
13.23	13.03	13.17	–	–	–	23.74	24.8	24.45
16.85	17.38	16.72	–	–	–	27.16	27.14	26.83
20.75	20.77	20.47	–	37.96	–	–	37.76	37.39
24.31	24.6	24.02	–	–	–	–	–	–
29.12	28.12	28	–	–	–	–	–	–
33.97	33.87	34.8	–	–	–	–	–	–

## Quantification and statistical analysis

Before calculation, by substituting the average Ct value of the virus samples for each dilution into the standard curve equation, the copy number of the diluted virus samples can be obtained.

Examplestandardcurveequation:copies=exp((Ct-40.397)/-1.59754)

The genome number of diluted virus is equal to the copy number. The titer (genomes/μL) is equal to the genome number multiplied by the dilution factor. The titer (genomes/mL) is equal to the titer (genomes/μL) multiplied by 1000 (see Table 17 below).

**Table 17:** Example process of RVΔG-4EGFP rabies virus titer calculation

Dilution factor of total virus	Ct value 1	Ct value 2	Ct value 3	Ct average	Copies	Genomes	Titer (genomes/μL)	Titer (genomes/mL)	Average titer (genomes/mL)
50x∗	18.64	18.58	18.85	18.69	7.96E+05	3.98E+07	3.98E+07	3.98E+10	2.43E+10
500x	23.74	24.8	24.45	24.33	2.33E+04	1.17E+07	1.17E+07	1.17E+10	
5000x	27.16	27.14	26.83	27.04	4.27E+03	2.13E+07	2.13E+07	2.13E+10	

Note: Here is the calculation process of the dilution factor. From 5 μL virus we get 32 μL RNA. We use 16 μL RNA for DNase I treatment (50 μL reaction mix). From 50 μL mix, we pick 8 μL for cDNA generation (20 μL reaction mix).To make it easy for adding the sample using multipipette we first dilute the cDNA to 5-fold (10 μL cDNA diluted to total 50 μL) and then add 5 μL diluted cDNA into qPCR reaction mix as the first dilution series, which means that we pick 1/50 μL ( 5∗16/32∗8/50∗10/20∗5/50 ) of the virus for qPCR test. As for the next two dilution series, we make 10-fold and 100-fold dilution separately compare to the first diluted cDNA sample.

## Limitations

### Detection of G in RVΔG virus or L in RVΔL virus

In the actual virus titer detection experiment, we detect the trace presence of G in the RVΔG virus, with the usual Ct value above 30. Since DNase I treatment has been performed, it is considered that its source may be the cell line’s residual genomic DNA. Since there is a small sequence difference at the C-terminal between the B19G used to construct the cell line and the G used for virus rescue, therefore, differential primers are designed to identify the source of G. The PCR results confirmed our hypothesis (shown in supplementary Figure 1, see it in Mendeley Data: https://doi.org/10.17632/kyrmm37m5v.1).

### Differences in titer values measured by FACS and RT-qPCR

We compared the differences of RVΔG and RVΔL rabies virus titer value measured by FACS and RT-qPCR. The recombinant rabies virus titer value obtained through the calculation of FACS is slightly higher than RT-qPCR in our experiment (Figures 2H and 2I). We suppose that this may be caused by the certain efficiency of reverse transcription and the sensitivity of the qPCR primers.

### Titration by RT-qPCR can’t distinguish the remaining B19G enveloped rabies virus in EnvA enveloped rabies virus

We can use TVA950-expressed cell line when titrating by FACS to detect EnvA enveloped rabies virus and at the same time use wild-type HEK-293T cell line to detect remaining B19G enveloped rabies virus. However, RT-qPCR detect rabies virus genome copy number to tell the titer, so it is not possible to tell the difference between B19G or EnvA enveloped rabies virus.

## Troubleshooting

### Problem 1

The virus rescue efficiency is low (related to Step 2 in before you begin).

### Potential solution

The cell density needs to be 80% cell density before you start plasmid transfection for virus rescue. The virus titer will be low if the cell density is below or over 80%.

### Problem 2

Detection of G in pRVΔG standards or L in pRVΔL standards (related to Steps 1-5 in step-by-step method details).

After preparation of standards storage, you should draw a standard curve as quality control. If you detect G in pRVΔG standards or L in pRVΔL standards, it means that there is plasmid contamination in your standards. Consider using the following solution to avoid this problem.

### Potential solution

DON’T prepare pRVΔG-4mcherry and pRVΔL-5Cre plasmids at the same day to prevent detection of L in pRVΔL-5Cre standards or G in pRVΔG-4mcherry standards.

### Problem 3

The R^2^ value is away from one (related to Step 27 in step-by-step method details).

You need to draw standard curve every time when you perform the qPCR titration experiment. Every time you get a standard curve equation with slightly different. The R2 value should be nearly one for a high-quality curve.

### Potential solution

Every STD sample must be mixed thoroughly before taking out for the preparation of the next dilution of the sample. If not, you will get a standard curve with its R2 value away from 1, the standard curve can’t be used for virus titer calculation. We offer three qualified standard curves for RVΔG, RVΔL and CVS-N2CΔG respectively (Figures 2B, 2D, and 2E). Also, we offer one standard curve that doesn’t qualify to be used for subsequent analysis in Figure 2C.

### Problem 4

The Ct value is different if you perform the qPCR experiment at different day (related to Step 27 in Step- by-step method details). If the virus cDNA is stored in −20°C, even if it is only stored overnight, the detected virus titer will decrease.

### Potential solution

Consider doing the subsequent qPCR steps as soon as you obtain the cDNA, or you can keep consistency in your experiment for the time between qPCR experiment and cDNA acquisition.

### Problem 5

Forget to consider the dilution ratio during calculation of the virus titer (related to Steps 27 in Step- by-step method details).

### Potential solution

Record the partition of sample you’ve taken during each step from RNA extraction to qPCR steps. You should multiply the ratio when calculate the final virus titer.

## Resource availability

### Lead contact

Further information and requests for resources and reagents should be directed to and will be fulfilled by the lead contact, Lei Jin (jinlei@lglab.ac.cn).

### Technical contact

Technical questions on executing this protocol should be directed to and will be answered by the technical contact, He Zhang (zhanghe@lglab.ac.cn).

### Materials availability

Please contact Lei Jin (jinlei@lglab.ac.cn) if need the cell lines or a protocol for making any cell lines in the manuscript.

### Data and code availability

Original data for all the figures and the supplementary figure 1 and files 1-3 in the paper is available [Mendeley Data: https://doi.org/10.17632/kyrmm37m5v.1].

## Acknowledgments

This work was supported by Lingang Laboratory (startup fund to L.J.) and by grants to H.Z. from the China Postdoctoral Science Foundation (2023M741508). We thank device support from the laboratory animal center, gene-editing core facility, and optical imaging platform in Shanghai Center for Brain Science and Brain-Inspired Technology as well as Lingang Laboratory. The graphical abstract was created with BioRender.com. L.J. appreciates all team members for their extraordinary work and also thanks his adorable son for letting him finish this work.

## Author contributions

Conceptualization, H.Z. and L.J.; methodology, H.Z. and L.J.; investigation, H.Z., X.Gao., X.W., X.Ge., and M.S.; writing – original draft, H.Z., X.Gao., X.W., X.Ge., and M.S.; writing – review and editing, H.Z. and L.J.; funding acquisition, L.J. and H.Z.; resources, X.Z.; supervision, L.J.

## Declaration of interests

The authors declare no competing interests.
